# Proteomic shifts in multi-species oral biofilms caused by *Anaeroglobus geminatus*

**DOI:** 10.1038/s41598-017-04594-9

**Published:** 2017-06-30

**Authors:** Kai Bao, Nagihan Bostanci, Thomas Thurnheer, Georgios N. Belibasakis

**Affiliations:** 1 Division of Oral Microbiology and Immunology, Institute of Oral Biology, Center of Dental Medicine, University of Zürich, Zürich, Switzerland; 2 Department of Dental Medicine, Karolinska Institute, Stockholm, Sweden

## Abstract

*Anaeroglobus geminatus* is a relatively newly discovered putative pathogen, with a potential role in the microbial shift associated with periodontitis, a disease that causes inflammatory destruction of the periodontal tissues, and eventually tooth loss. This study aimed to introduce *A. geminatus* into a polymicrobial biofilm model of relevance to periodontitis, and monitor the proteomic responses exerted to the rest of the biofilm community. *A. geminatus* was grown together with another 10-species in a well-established “subgingival” *in vitro* biofilm model. Its effects on the other species were quantitatively evaluated by qPCR and label-free proteomics. *A. geminatus* caused a significant increase in *P. intermedia* numbers, but not the other species in the biofilm. Whole cell proteome profiling of the biofilms by LC-MS/MS identified a total of 3213 proteins. Label-free quantitative proteomics revealed that 187 proteins belonging to the other 10 species were differentially abundant when *A. geminatus* was present in the biofilm. The species with most up-regulated and down-regulated proteins were *P. intermedia* and *S. oralis*, respectively. Regulated proteins were of primarily of ribosomal origin, and other affected categories involved proteolysis, carbon metabolism and iron transport. In conclusion, *A. geminatus* can be successfully grown in a polymicrobial biofilm community, causing quantitative proteomic shifts commensurate with increased virulence properties.

## Introduction

Periodontal infection is one of the major causes for tooth loss, with an estimated prevalence of 50% in the adult population of the USA^1^. Microbial biofilms growing on the tooth surface and consisting of a multitude of different species^2^ are the etiological factor of the initiation of the disease^3^. Within biofilms, oral microbes display increased virulence and become more resistant to antibiotics and to the immune system, compared to living in planktonic form^4, 5^ Despite the deleterious role of oral biofilms in periodontal infection, commensal oral bacteria actually support the maintenance of a healthy periodontium, by minimally priming the immune responses to a readiness level^6^. Switch of a microbial community from a commensal to a “dysbiotic” one may result in exacerbated tissue-destructive inflammation. This requires the synergy between metabolically compatible microorganisms within the biofilm, and entails an imbalanced relationship with the host immune response^7, 8^. Thus, understanding the role and behaviour of different microbes as counterparts of a biofilm community is essential for understanding the microbiome shifts that act as a driving force for the establishment of periodontal infection.

A large number of microorganisms have been identified as candidates in the etiology of periodontal infections, particularly with the development of novel molecular diagnostic technologies^9^. *Anaeroglobus geminatus* was not reported until 2002, when it was directly characterized as a strictly anaerobic gram-negative coccus^10^ belonging to the family *Veillonellaceae* and closely related to *Megasphaera* spp^11^. The prevalence of this species was found to be increased in patients with chronic periodontitis^12^ and apical periodontitis^13^, correlating with low bleeding on probing scores (indicative of gingival inflammation)^14^, and higher proportion of colonization of subgingival sites in chronic and aggressive periodontitis^15^. These clinical observations indicate that *A. geminatus* is associated with periodontal diseases, at least under certain circumstances. Yet, the influence of *A. geminatus* in the dynamic shifts occurring in an oral biofilm community has never been investigated.

The preamble for oral biofilm formation is the attachment of bacteria on the tooth surface^16^, followed by the sequential attachment and growth of further species, resulting in a complex microbial community with established inter-species communication^17^. Therefore, polymicrobial *in vitro* biofilm models are a useful resource to study communication and behavioural interactions between species. For instance, by using such models it was determined that BspA proteins of *Tannerella forsythia* were not imperative for biofilm formation when grown together with *P. gingivalis*, despite their positive effect on co-aggregation^18^. In addition, it was also determined that gingipains, a set of important virulence factors of *P. gingivalis*, regulate the behaviour of other species in biofilms. The lysine-specific gingipain favoured the growth of *T. forsythia* ^19^, while the Arginine-gingipain supported the growth of *Treponema denticola* in a dual-species^20^, as well as a 10-species biofilm^19^. This 10-species biofilm model consists of bacteria commonly represented in subgingival biofilms associated with periodontitis, and has proved to be a useful model in evaluating the effects of individual species within the oral biofilm community. As such, the model has recently been used to evaluate the role of *Aggegatibacter actinomycetemcomitans* in the biofilm community, by developing a label-free quantification proteomics workflow to study the internally regulated proteins^21^. With this state-of-art liquid chromatography-tandem mass spectrometry (LC-MS/MS) technology, it is possible to identify a plethora of proteins in a single run. In conjunction with label-free quantification, it is further possible to quantify the regulation of these identified proteins. In the present study we used this biofilm model and proteomic approach, with the aim to evaluate the changes brought by *A. geminatus* to other species and chart the protein regulatory patterns in the biofilm, induced by its presence. This is the first molecular approach to understand the mechanisms of actions of *A. geminatus* within biofilms, with potential implications in periodontal infections.

## Results

### Integration of *A. geminatus* into the biofilm increased the growth of *Prevotella intermedia*

**Figure 1:**
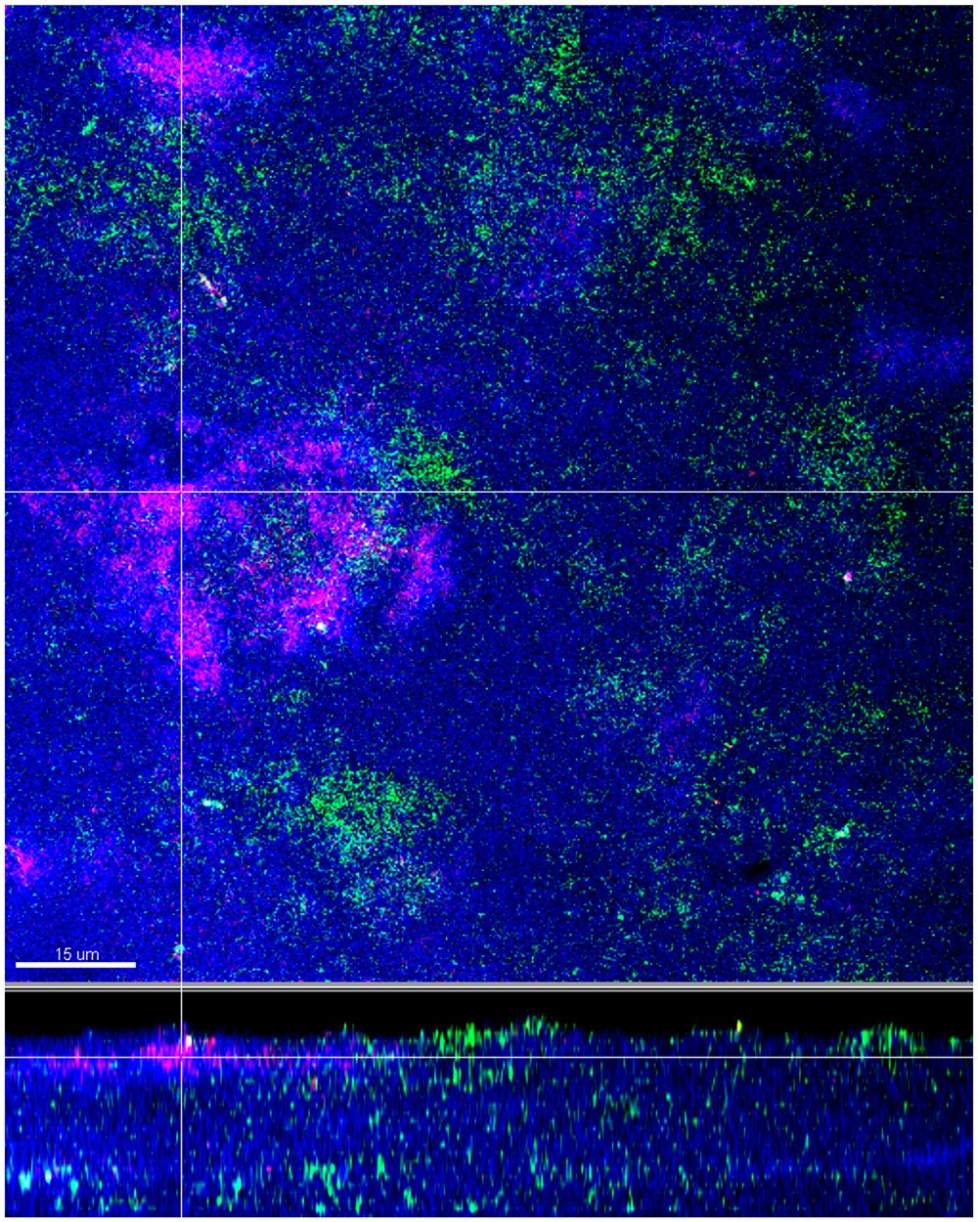
Localisation of *A. geminatus* within the biofilms. Confocal laser scanning microscopy images of fluorescence *in situ* hybridization (FISH) -stained 11-species biofilm. Bacteria appear blue due to DNA-staining using Syto 59. Due to FISH staining *A. geminatus* and *V. dispar* appear red and green, respectively. The biofilm base in the cross-sections is directed towards the top view. Scale bar: 15 µm.

The biofilms were cultivated anaerobically for 64 h before being harvested. The localization of *A. geminatus* within the biofilm structure was determined by combination of fluorescence *in situ* hybridization (FISH) staining and visualization by confocal laser scanning microscopy (CLSM). It was evident that *A. geminatus* was mostly clustered in the form of aggregates and mainly distributed on the “inner” biofilm side closer to the hydroxyapatite (HA) disc surface (Fig. 1). From suspended biofilm, numbers of each species within the biofilms were quantified by quantitative real-time polymerase chain reaction (qPCR). A statistically significant difference between biofilm groups was only observed in the case of *P. intermedia* (*p* = 0.0165), which was increased in the presence of *A. geminatus* (i.e. 11 species biofilm), while no significant changes in numbers were detected regarding the other species (Table 1).

**Table 1:** Quantitative composition of the 11-species or 10-species biofilm after 64 h cultivation.

	11-species biofilm		10-species biofilm		
	Mean^1^	SD	Mean^1^	SD	P-value
*A. geminatus*	1.42E + 09	2.81E + 08			–
*A. oris*	1.62E + 08	1.90E + 07	2.05E + 08	7.05E + 07	0.4002
*C. rectus*	3.19E + 07	5.69E + 06	2.89E + 07	1.64E + 06	0.4571
*F. nucleatum*	2.08E + 09	4.08E + 08	1.52E + 09	5.44E + 08	0.2343
*P. gingivalis*	2.26E + 08	4.06E + 07	1.07E + 08	6.94E + 07	0.0777
*P. intermedia*	3.36E + 09	7.60E + 08	1.96E + 08	1.32E + 08	0.0165*
*S. anginosus*	5.79E + 08	9.01E + 07	1.09E + 09	6.91E + 08	0.3284
*S. oralis*	1.33E + 09	3.36E + 08	9.90E + 08	4.76E + 08	0.3704
*T. denticola*	9.28E + 06	6.08E + 06	3.70E + 06	2.44E + 06	0.2493
*T. forsythia*	1.61E + 08	1.00E + 07	6.38E + 07	5.07E + 07	0.0750
*V. dispar*	1.64E + 09	3.21E + 08	1.25E + 09	2.26E + 08	0.1715

^1^Quantification was performed by qPCR for each species, per HA disc. The data is expressed as the bacterial mean counts ± standard deviation (SD) from three biological replicates.

Student t test: *p-value < 0.05.

### *Anaeroglobus geminatus* alters the proteomic profile of the biofilm

**Figure 2:**
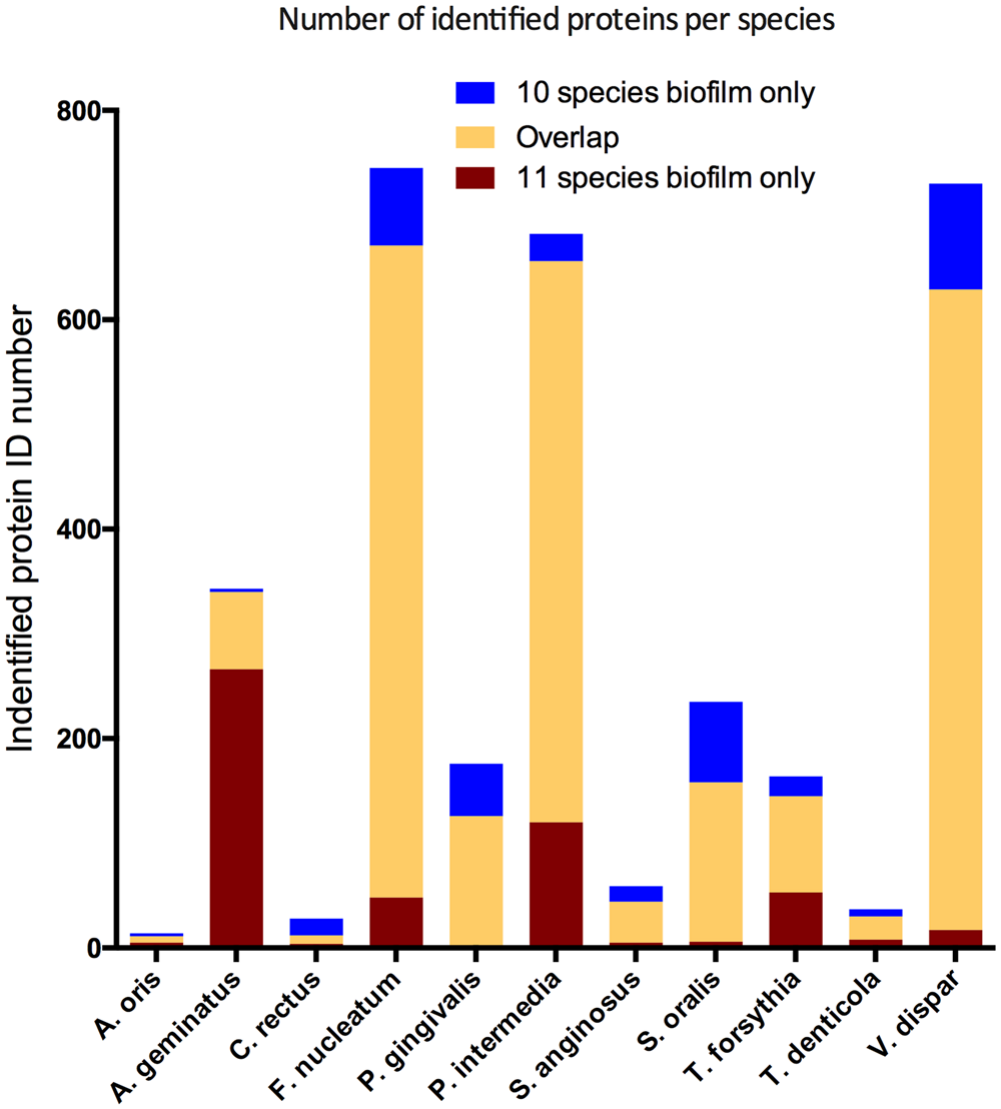
Number of identified proteins from each species. Identified proteins are shown based on whether they were uniquely identified in the 10 species biofilm alone (blue), 11 species biofilm alone (yellow), or in both groups (red).

Proteins were accepted only if at least two unique peptides were present for the global proteomic analyses performed. In all, a total of 2822 and 2679 bacterial proteins with a corresponding false discovery rate (FDR) of 1.6% and 1.3% were identified in the 11-species and 10-species biofilms, respectively (Table S1). Of those, there was a considerable overlap of 2288 proteins in both biofilms. Yet, 534 proteins were exclusively identified in the biofilms where *A. geminatus* was present (11 species), while 391 proteins were exclusively identified in the biofilm where *A. geminatus* was absent (10 species). For *F. nucleatum* and *V. dispar*, more than 80% of their proteins were commonly identified in both biofilm variants, whereas only a small number of *C. rectus* and *A. oris* proteins were identified (Fig. 2).

**Figure 3:**
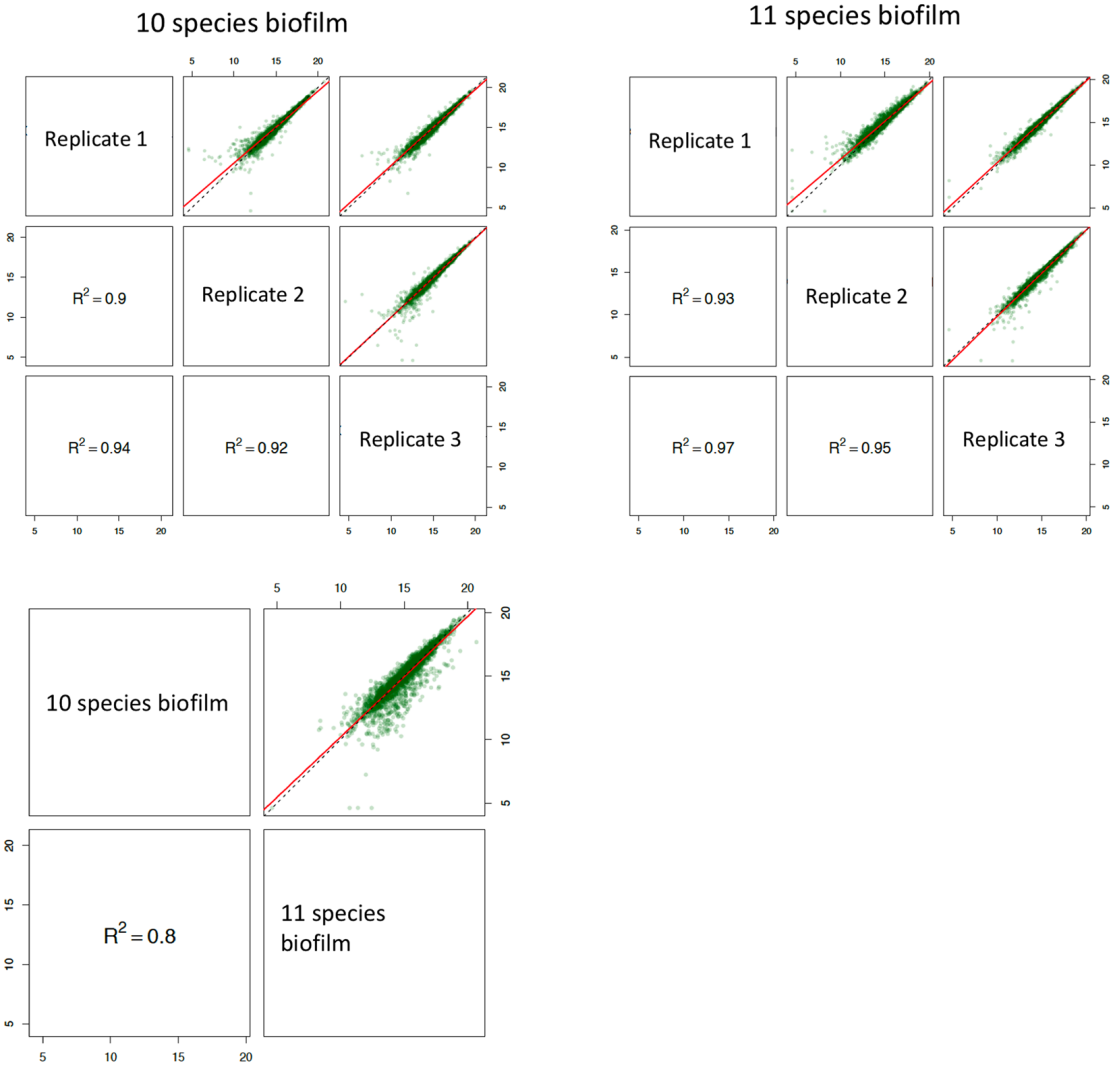
Quality control of the label-free quantitation data. Squared Pearson correlation coefficients of integrated peptide feature intensities are displayed for the comparisons within (n = 3) and between the two biofilm groups. The linear regressions of the integrated peptide feature intensities in different experimental group conditions are indicated in red, whereas the dashed lines correspond to direct proportionality.

**Figure 4:**
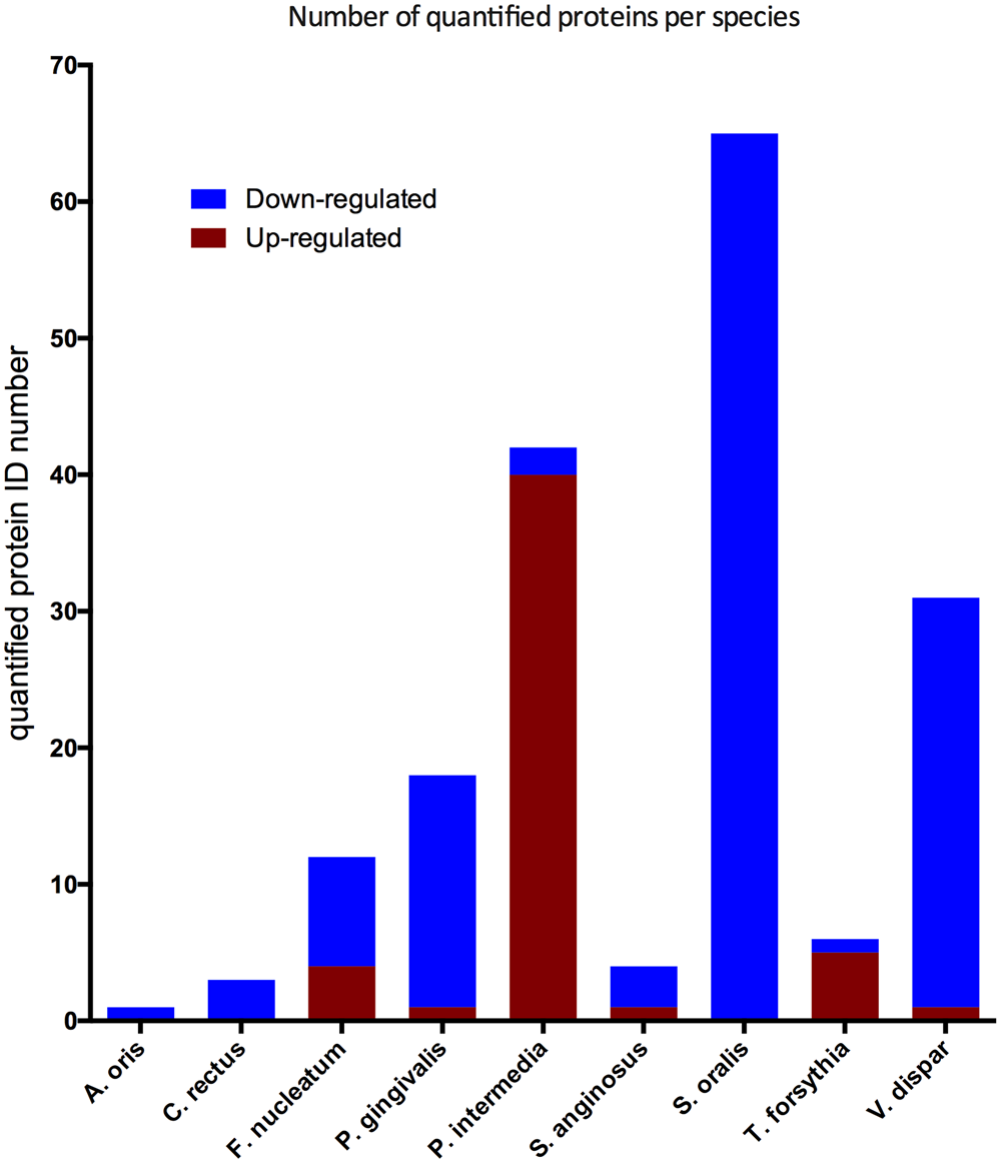
Number of differentially regulated proteins from each species. Regulation trends of label-free quantified proteins are shown based on whether they are down- (blue) or up- (red) regulated in relation to the present of *A. geminatus* in the biofilm. A significant (p < 0.05) difference of 2-fold in protein levels was defined as “regulation”.

The identified proteins were further analysed by the Progenesis software for label-free quantification. Squared Pearson correlations coefficients (R^2^) were calculated to measure the reproducibility between samples by the SafeQuant software. It was found that R^2^ ranged between 0.9–0.92 among the 10 species biofilm group, and between 0.93–0.95 among the 11 species biofilm group, while this value was 0.8 in the comparison between these two biofilm variants (Fig. 3). Proteins that had log_2_ ratios difference of more than 1-log and q < 0.05 between biofilm groups were considered as truly regulated ones. Hence, a total of 374 bacterial proteins (including 192 *A. geminatus* proteins in the respective biofilm group), were identified as regulated ones, for subsequent label-free quantification (Table S2). Among the 182 proteins from the remaining 10 bacterial species, 52 were up-regulated in the presence of *A. geminatus*, whereas 130 were accordingly down-regulated (Fig. 4). The abundance of several *P. intermedia* proteins was increased, perhaps not surprisingly, since the absolute numbers of this species in the biofilm were significantly elevated in the presence of *A. geminatus*. On the contrary, several *S. oralis* proteins were down-regulated when *A. geminatus* was present in the biofilm (Fig. 4). Interestingly, around 20% of the regulated proteins were ribosomal ones, mainly belonging to *P. intermedia* and *S. oralis*. Among the 52 upregulated proteins, 17 were ribosomal (16 from *P. intermedia* and 1 from *T. forsythia*). Among the 130 downregulated proteins, 19 were ribosomal (18 from *S. oralis* and 1 from *P. gingivalis*) (Table S2). Of note, with the particular analytic strategy approach used, none of the *T. denticola* proteins were eligible for quantification.

### *Anaeroglobus geminatus* regulates biological pathways in the biofilm

**Figure 5:**
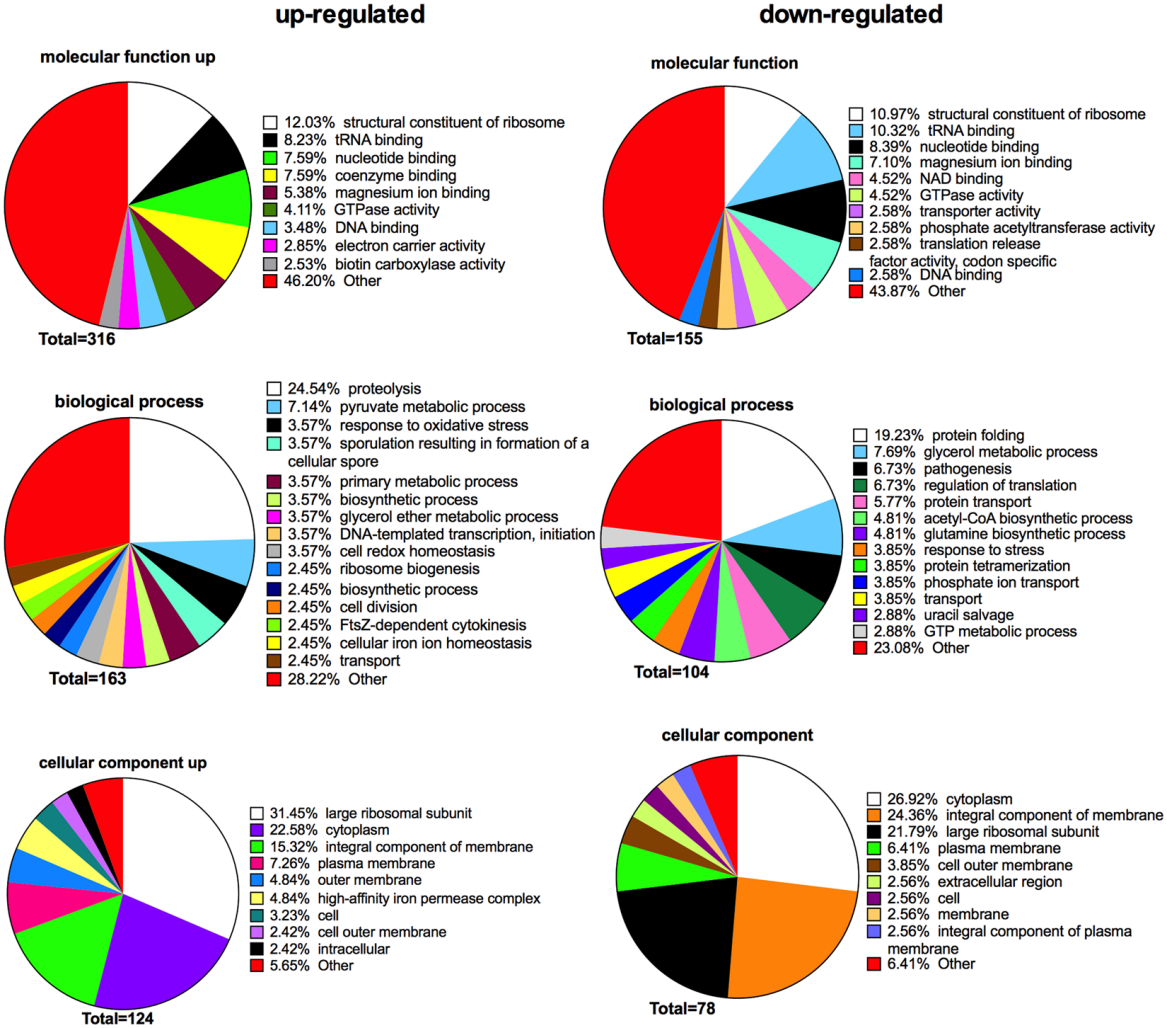
Annotation of regulated bacterial protein functions by Gene Ontology (GO) terms enrichment. The comparison was performed between the two biofilm variants. The GO terms from all regulated bacterial functions were categorized into three categories: a) biological process, b) molecular function, and c) cellular component, as displayed in the pie charts. These categories are displayed as “upregulated” or “downregulated” in relation to the presence of *A. geminatus* in the biofilm. The numbers of GO terms for each of the three categories are shown, whereas the proportion of each specific subcategory is also provided. Subcategories with GO terms less than 2% are classified as “other”.

The gene ontology (GO) terms of all regulated proteins were collectively pooled to decipher the regulatory effects of *A. geminatus* on biological pathways in the biofilm (Fig. 5). In this whole-biofilm-function approach, regulated proteins from all 11 species were taken into consideration. GO terms were terminologically structured into three domains: a) molecular function, b) biological process, and c) cellular component. In brief, 316, 163, and 124 GO terms for molecular function, biological process and cellular component, respectively, were generated from the proteins that were upregulated in the presence of *A. geminatus*. Accordingly, 155, 104, and 78 GO terms for molecular function, biological process, and cellular component, respectively, were generated from the proteins that were downregulated in the presence of *A. geminatus*. This collection of GO terms indicated that the most frequently regulated molecular function was “structural constituent of ribosome”. Many top enriched GO terms from other domains were also ascribed ribosome-related functions. For instance, the most frequent “cellular component” GO term among upregulated proteins, indicated that many proteins belonged to the “large ribosomal subunit”, while the most frequent “molecular function” GO term among downregulated proteins was “structural constituent of ribosome”. Interestingly, the most upregulated biological process was “proteolysis”, whereas the most downregulated one was “protein folding”. The former may imply that *A. geminatus* could trigger proteolytic events by other species present in the biofilm.

## Discussion

There is an increasing demand to elucidate the microbial etiology of periodontal infections in the more “holistic” context of oral biofilms^19, 22^, rather than individual “putative pathogens”. This holistic view is further complemented by the application of “omic” technologies that enable the simultaneous screening of a vast number of outputs^23, 24^. In this light, the present *in vitro* study utilized a proteomic platform to monitor the behaviour and effects of *A. geminatus* in a multi-species biofilm environment, an approach that we recently used to thoroughly studied microorganism *Aggregatibacter actinomycetemcomitans* ^25^.

Despite circumstantial evidence on the detection of *A. geminatus* in periodontitis-associated biofilms^12–14^, there has been as-yet no mechanistic evidence of its potential role and effects within a complex subgingival biofilm community. This was addressed in the present study. It was found that the presence of *A. geminatus* in the biofilm did not significantly perturb the growth of other species in the biofilm, but potentiated the growth of *P. intermedia*, denoting a synergistic association worth investigating further.

Proteomic analysis by application of LC-MS-MS revealed that *A. geminatus* actually affected the protein-regulatory behaviour of a number of other species. As such, many proteins from *P. gingivalis*, *S. oralis* and *V. dispar* were not detectable when *A. geminatus* was present in the biofilms, while the opposite was the case for *P. intermedia* and *T. forsythia*. Considering that peptides are indicators for protein abundance^26^, this finding denotes that *A. geminatus* causes shifts in the protein expression of others species in the biofilm. Furthermore, label-free quantification enables a more accurate evaluation of the regulated proteins in this *in vitro* model.

Among the constituent bacteria of the biofilm, *P. intermedia* appears to be the species most affected by *A. geminatus*, based on the changes caused on its cell and protein numbers. *Prevotella intermedia* is a frequent find in biofilms from sites with periodontal disease^27^, and its presence positively correlates Interleukin (IL)-1β in gingival crevicular fluid^28^, clinical attachment loss^29^, bleeding on probing^30^, as well as periodontal inflammation during pregnancy^31^. Besides, *P. intermedia* is regarded as one of the most prevalent antibiotic-resistant species among bacteria isolated from primary dental infections^32–34^, and its resistance may be further strengthened when growing within the biofilm^35^. Interestingly, using a similar experimental model as in this study, we have shown that the protein profiles of *P. intermedia* were also significantly regulated when integrating *A. actinomycetemcomitans*, another putative periodontal pathogen, into this multi-species biofilm^21^. An interesting functional property of *P. intermedia* is its ability to synergise together with *P. gingivalis* for heme acquisition^36^, which might affect the iron intake and consumption by the biofilm, to the growth benefit of both these species.

*Streptococcus oralis* is another species that was strongly effected by *A. geminatus*. Contrary to *P. intermedia*, this bacterial species down-regulated many proteins without a significant change in cell numbers, when *A. geminatus* was present in the biofilm. Interestingly, in similar biofilm models, *S. oralis* and *P. intermedia* protein expressions also showed a reverse regulatory patterns in the presence of *A. actinomycetemcomitans* ^21^ or of gingival organotypic tissue^37^. Here, more *S. oralis* proteins were identified and up-regulated in the presence of *A. geminatus*, while more *P. intermedia* proteins were identified and up-regulated in its absence, despite that no significant changes in individual bacterial numbers were found between the two biofilm groups. The interrelation between the two species is also revealed by the fact that removal of streptococci from the 10-species biofilm model resulted in formation of long chains by *P. intermedia* that resembled streptococci^38^, in line with the notion that *P. intermedia* and streptococci display differential growth patterns during the various stages of biofilm development^3^. The findings of the present study using proteomics may also confirm a competitive trend between *S. oralis* and *P. intermedia*, in this case mediated by *A. geminatus* in the biofilm.

GO terms from all the regulated proteins were enriched and used to give an overview of functional changes among the two biofilm variants. The terms of these proteins were divided into three different categories: molecular function, biological process and cellular component, based on their default classification. In the “molecular function” category, six of the ten terms from up-regulated proteins were also be found among down-regulated proteins. Moreover, the three most abundant ontologies were all ribosomal-related and appeared in both regulated groups in exactly the same order. These large numbers of ribosomal-related functions were also reflected on the biological process with the most common terms being “proteolysis” and “protein folding”, for the up-regulated and down-regulated group, respectively. On the other hand, both parts included many terms that did not reach even 2% of the total enriched functions. Hence, this resulted in 46.20% of the up-regulated and 43.87% of the down-regulated proteins, respectively, belonging to the grouping “other”. In summary, the pie chart overview of GO terms indicated that *A. geminatus* shifted ribosomal protein expressions, contributing to newly synthesized proteins with diverse functions.

Expression of ribosomal proteins is typically associated with bacterial growth phase. This may be consistent with the present observation on the increased number of *P. intermedia* and overweighing up-regulation of its ribosomal proteins in the presence of *A. geminatus*. It is known that ribosomal protein abundance could be adapted according to environmental conditions or stresses^39^. For instance, studies on *Escherichia coli* have shown shifts in ribosomal protein expression, under diverse environmental stresses^40^ and growth phases^41, 42^. Therefore, the ribosome is an important protein translation factor rather than a mere protein synthesis machine^43^. This is probably the case for *S. oralis*, as the down-regulated proteins in the presence of *A. geminatus* are predominantly ribosomal ones. Bacterial ribosomal subunits are targets for many antibiotics, such as tetracycline^44^, azithromycin^45^ and different other aminoglycosides^46^. Therefore, another important aspect on the ribosomal proteins is their association with antibiotic resistance^47–49^. Although the contribution of ribosomal proteins to antibiotic resistances of single bacterial species is heavily discussed^39^, there is lack of such knowledge in terms of whole biofilm communities. The regulatory effect of *A. geminatus* on ribosomal protein expression in the biofilm could indicate a role in antibiotic resistance, which, however, needs to be validated with functional assays.

*Anaeroglobus geminatus* may also affect the carbon metabolic pathways of the whole biofilm community. In the biological process category, the most up-regulated metabolic proteins belonged to the “pyruvate metabolic process” group, whereas the most down-regulated ones belonged to the “glycerol metabolic process” group. Interestingly, both terms are the second most frequent GO terms, adjacent to the ribosomal-related functions on each group. Metabolic regulation and microbial virulence can be associated properties in many species^50–52^, and therefore one could postulate that the carbon metabolic pathways triggered by the presence of *A. geminatus* may contribute to the overall virulence of the biofilm.

The cellular component categories for the most up- or down- regulated proteins were quite similar, with most of the GO terms appearing in both groups and sharing even similar ranks. The finding of main interest was that more proteins of the “high-affinity iron permease complex” category were present in the up-regulated group. Iron binding in the closed environment of the periodontal pocket is always associated with digestion of host iron-containing proteins^53–55^, which can be achieved by anaerobic and proteolytic bacteria such as *P. gingivalis* and *P. internedia*. This function is related to bacterial virulence and in line with the previous hypothesis that *A. geminatus* might contribute to the overall virulence of the oral biofilm.

In conclusion, this study succeeded in incorporating an isolate of the barely studied *A. geminatus* species, into a multi-species biofilm of relevance to periodontal disease. Moreover, it evaluated the deep proteomic changes that this newly introduced species exerted to the whole biofilm community. It significantly boosted the growth of *P. intermedia* in the biofilm, but had little effect on the other species. Yet *A. geminatus* caused shifts in the protein expression profile of the whole biofilm community, with particular effects on ribosomal proteins, proteolysis, carbon metabolic processes and iron transport. Collectively, these changes may imply a role of *A. geminatus* in increasing the virulence of a subgingival biofilm, a property which, however, needs to be tested functionally.

## Methods

### Multispecies biofilm formation and harvesting

The 11 species used to establish a “subgingival” biofilm *in vitro* included *Actinomyces oris* (OMZ 745), *Anaeroglobus geminatus* CCUG 44773 (OMZ1129), *Campylobacter rectus* (OMZ 398), *Fusobacterium nucleatum subsp. nucleatum* (OMZ 598), *Streptococcus oralis SK248* (OMZ 607), *Treponema denticola* ATCC 35405 T (OMZ 661), *Streptococcus anginosus* ATCC 9895 (OMZ 871), *Tannerella forsythia* (OMZ 1047), *Prevotella intermedia* ATCC 25611 T (OMZ 278), *Porphyromonas gingivalis* W50 (OMZ 308) and *Veillonella dispar* ATCC 17748 T (OMZ 493). The relative effects of *A. geminatus* in the biofilm community were evaluated by growing in parallel, and comparing to a 10-species biofilm variant in which *A. geminatus* was absent. Biofilms were grown according to our previously described standard model^15, 19, 21, 37, 38, 56, 57^. Briefly, 200 μl of bacterial suspensions from each strain containing equal densities (OD_550_ = 1.0) were seeded onto the HA discs and incubated anaerobically for 64 h. The biofilm discs were dip-washed in 0.9% w/v of NaCl at 16 h, 20 h, 24 h, 40 h, 44 h, 48 h and 64 h, with the medium replenished at 16 h and 40 h during the incubation period. Both biofilm variants (11- and 10-species) were represented in three biological replicates. The formed biofilms were then either fixed in 4% paraformaldehyde for CLSM analysis or sonicated and collected in a suspension of 0.9% w/v NaCl for downstream qPCR and proteomic analysis.

### Confocal laser scanning microscopy and image analysis

The paraformaldehyde-fixed biofilms were stained by FISH and subjected to CLSM for visually investigating the structure of the biofilm. The cy3- labelled 16 S rRNA oligonucleotide probe MegAg1147 was used to detect *A. geminatus* (sequence from 50 to 30: TGCGGCWGTCTCTCCTGA, formamide concentration: 40%, NaCl concentration in wash buffer: 46 mM), whereas the FAM-labelled 16 S rRNA oligonucleotide probe VEI217 was used to detect the close related *V. dispar* (sequence from 50 to 30: AATCCCCTCCTTCAGTGA, formamide concentration: 40%, NaCl concentration in wash buffer: 46 mM)^58^. Syto 59 was used to counter stain the biofilm following the protocol described before^19^. A Leica sp5 confocal microscope (Leica Microsystems) was used for visualization. The microscope filters were set to 500–540 nm to detect FAM, to 570–630 nm for Cy3, and 660–710 for Syto 59. All images were captured with a 63 × objective (glycerol immersion, NA 1.3) and processed using Imaris 7.4.0 software (Bitplane) to reconstruct the biofilm.

### Quantitative real-time polymerase chain reaction

A qPCR assay was performed to quantify the numbers of individual species in the biofilm, using the bacterial DNA extracts from the collected biofilm suspension. The bacterial DNA was extracted using the GenElute bacterial genomic DNA kit (Sigma-Aldrich) as described previously^15^. The qPCR assays were operated using SYBR Green PCR Master Mix (Life Technologies) on a StepOnePlus Real-Time PCR System (Applied Biosystems). Microbial DNA qPCR Assay for *A. geminatus* (Qiagen) was used to detect its target species, while other species-specific 16 S rRNA gene (Table 2) primers for the qPCR were designed using online NCBI/Primer-BLAST tool (http://www.ncbi.nlm.nih.gov/tools/primer-blast) as reported previously^59^. The bacterial numbers were calculated using standard curves generated with DNA extracted from planktonic bacterial cell cultures, as described previously^59^.

**Table 2:** Primer sequences and properties.

Organism	Sequence (5′→3′)	Strand on template	T_m_ (°C)	Reference
*S. anginosus*	ACCAGGTCTTGACATCCCGATGCTA	+	59.25	
	CCATGCACCACCTGTCACCGA	−	59.04	
*S. oralis*	ACCAGGTCTTGACATCCCTCTGACC	+	59.42	
	ACCACCTGTCACCTCTGTCCCG	−	59.85	
*A. oris*	GCCTGTCCCTTTGTGGGTGGG	+	59.57	59
	GCGGCTGCTGGCACGTAGTT	−	60.32	
*V. dispar*	CCCGGGCCTTGTACACACCG	+	59.7	59
	CCCACCGGCTTTGGGCACTT	−	59.83	
*F. nucleatum*	CGCCCGTCACACCACGAGA	+	59.04	59
	ACACCCTCGGAACATCCCTCCTTAC	−	59.48	
*C. rectus*	TCACCGCCCGTCACACCATG	+	59.35	59
	CCGGTTTGGTATTTGGGCTTCGAGT	−	59.5	
*P. intermedia*	GCGTGCAGATTGACGGCCCTAT	+	59.61	59
	GGCACACGTGCCCGCTTTACT	−	60.24	
*P. gingivalis*	GCGAGAGCCTGAACCAGCCA	+	59.07	59
	ACTCGTATCGCCCGTTATTCCCGTA	−	59.44	
*T. denticola*	TAAGGGACAGCTTGCTCACCCCTA	+	58.84	59
	CACCCACGCGTTACTCACCAGTC	−	59.76	
*T. forsythia*	CGATGATACGCGAGGAACCTTACCC	+	59.07	59
	CCGAAGGGAAGAAAGCTCTCACTCT	−	58.01	

*T*_m_, melting temperature.

### Biofilm protein extraction for proteomic analysis

For the downstream proteome analysis, protein extracts of the biofilm were performed, as previously described^21^. Briefly, pellets collected from the biofilms were suspended with 30 µl lysis buffer (4% w/v Sodium Dodecyl Sulfate (SDS), 0.1 mM dithiothreitol and 100 mM Tris-HCl pH 8.2), incubated at 95 °C for 5 min and chilled on ice for 45 sec. The mixtures were then sonicated three times, each for 3 min, 0.5 cycle for intervals and 65% ultrasonic amplitude. The samples were chilled on ice for 3 min after each ultra-sonication cycle. The concentrations of the extractions were measured using Qubit Protein Assay Kit (Life Technologies).

### Filter aided sample preparation (FASP) digestion and C18 clean up

Biofilm extracts were digested in the Microcon YM-30 centrifugal filter unit (Millipore) following the previously described protocol^21^. Briefly, 20 μg of extracts were mixed with 200 μl of urea buffer (8 M urea, 0.1 mM dithiothreitol in 100 mM Tris/HCl buffer (pH 8.2)) and loaded in the centrifugal filter unit. Samples were then denatured with additional 200 μl of urea buffer, alkylated with 100 μl of 0.05 M iodoacetamide, and washed three times by 100 μl 0.5 M NaCl. Then, 0.4 μg of trypsin (Promega) were diluted in 120 μl 0.05 M triethylammonium bicarbonate (TEAB) medium, and loaded on the filter unite for overnight digestion. Reagents from each of the above digestion steps were removed by centrifugation at 14,000 g for 20 min at 35 °C, before next ones were added. The digested peptide samples were collected by centrifugation under the same conditions, and acidified by trifluoroacetic acid to a final concentration of 0.5% w/v. The acidified samples were further diluted with 400 μl 3% acetonitrile in 0.1% TFA before C18 clean up.

Reverse phase cartridges Finisterre SPE C18 (Wicom International AG) were used to desalt the peptide mixture. In brief, each cartridge was wetted with 1 ml of 100% methanol, washed with 1 ml 60% acetonitrile (ACN) in 0.1% TFA, equilibrated with 2 ml of 3% ACN in 0.1% TFA, loaded acidified digested peptide samples, washed with 6 ml of 3% ACN in 0.1% TFA, and eluted with 0.5 ml of 60% ACN in 0.1% TFA. The desalted samples were dried in vacuum centrifuge and stored at −20 °C until further use.

### LC-MS/MS analysis

The desalted samples were solubilized with 30 μl of 3% ACN in 0.1% formic acid. In addition, a pooled sample of all subjects was prepared to serve as an alignment reference in the quantification analyses stage. All samples were analysed in an Orbitrap Fusion mass spectrometer (Thermo Fisher Scientific). Chromatographic separation of peptides was performed on an Easy nano-flow HPLC system (Thermo Fisher Scientific) coupled with a 15 cm long, 75 μm diameter, fused silica emitter that was packed with a ReproSil-Pur C18-AQ 120 A and 1.9 μm resin (Dr. Maisch HPLC GmbH). Peptides were separated with a linear gradient of acetonitrile/water (2 to 35% acetonitrile in 80 min), containing 0.1% formic acid, at a flow rate of 300 nl/min. A data-dependent strategy, with an automatic switch between MS and MS/MS using a top 12 method, was used to acquire mass spectra with a mass range of 300–1700 m/z. Higher energy collisional dissociation (HCD) peptide fragments, acquired at 28 normalized collision energy, were analyzed at high resolution in the Orbitrap.

### Database search analysis and protein identification

All raw files from LC-MS/MS were searched with Mascot (version 2.4.1) against a database containing 634157 sequence and 207,052,936 residues. This consists of *Homo sapiens* database (including isoforms) from Uniprot (release date 22 May 2014, containing 88,708 forward sequences and 88,708 reverse sequence as decoy), all proteins from the 11 bacterial species available in the NCBI database (release date 28 February 2014, containing 228,240 forward sequences and 228,240 reverse sequence as decoy), as well as a contaminants database with 261 sequences. The precursor ion tolerance was set to ± 10 ppm with a fragment-ion mass tolerance of ± 0.05 Da. The following search criteria were set: tryptic digests, max 2 missed cleavages for each peptide, iodoacetamide derivative as a fixed modification on cysteine, and oxidation or hydroxylation as variable modification on methionine residues. The Mascot research results of the biofilms were imported into Scaffold (version Scaffold_4.2.1, Proteome software) to validate the MS/MS-based peptide and protein identifications. The following protein thresholds were applied for the Scaffold research: 3.0% FDR for protein threshold, 2 minimal peptides, 1.0% FDR for peptide threshold. Only proteins with 2 or more unique peptides were accepted as “identified”.

### Label-free protein quantification and identification

The raw files from the mass spectrometer were loaded into Progenesis LC-MS software (Nonlinear Dynamics) for label-free quantification as described previously^21^. Briefly, only the non-conflicting peptide ions across the entire individual runs were automatic aligned with aligning reference (pooled sample from all conditions as a reference with the most features could visually be seen). From each Progenesis feature (default sensitivity in peak picking), a maximum of the top 6 tandem mass spectra were exported using charge deconvolution and deisotoping option and a maximum number of 200 peaks per MS/MS. The Mascot generic file (.mgf) was searched with Mascot using the parameters as it used for protein identification. The mascot result was then loaded into Scaffold_4.2.1. The following protein thresholds were applied for the Scaffold research: 1.0% FDR for protein threshold, 1 minimal peptides, 0.5% FDR for peptide threshold. The spectrum report was exported and loaded back into Progenesis. The missing values were handled in the software by use of co-detection, combined aggregate detection and accurate alignment approach. Normalization was kept with default settings by selecting “normalize to all compounds”. By default setting, the Progenesis software automatically selected one of the samples as a reference file, and then for each feature, it calculated a quantitative abundance ratio. Then, by use of log converted ratios and deviation outlier filtering method, a scaling factor was calculated between the samples. For further statistical validation, the calculated final protein reports were further imported to SafeQuant. Squared Pearson correlations coefficients (R^2^) were generated by SafeQuant to evaluate of the technical and biological reproducibility. Quantified proteins with significant q-value (q < 0.05), 2 or more unique peptides and absolute value of log2 ratios more than 1-log (2-fold), were considered as true regulated ones.

### Gene Ontology analysis

Regulated bacterial proteins were considered further in evaluating and characterizing the role of *A. geminatus* in the biofilm´s behaviour *in vitro*. Lists of Gene Ontology (GO) function were generated by Uniprot (release date 14th January 2016) using the Retrieve/ID Mapping function. The redundant GO terms were identified and removed based on analysis from REVIGO (release date 27th Jul 2015) using the “small (0.5)” as similarity option. Based on the lists of gene nomenclatures, these enriched GO terms were then manually summarized into three domains of ontology, namely a) molecular functional, b) biological process, and c) cellular component. The GO terms occurring with less than 2% of the whole GO content in each domain were clustered into the category “other”.

### Statistical analysis

The data derives from triplicate biofilms cultures in each group. The significance of differences in bacterial species levels between the two biofilm groups was calculated by Student´s t-test in Prism v.6 software (GraphPad, La Jolla California USA), using the logarithmically transformed bacterial numbers obtained by qPCR. Significance was considered at the level of P < 0.05.

## Electronic supplementary material

Supplementary Table 1

Supplementary Table 2
